# Epidemiology, clinical features, and classification of 3,404 patients with uveitis: Colombian Uveitis Multicenter Study (COL-UVEA)

**DOI:** 10.1007/s00417-024-06422-z

**Published:** 2024-03-06

**Authors:** Alejandra de-la-Torre, Germán Mejía-Salgado, Carlos Cifuentes-González, William Rojas-Carabali, Miguel Cuevas, Sandra García, Carlos M. Rangel, Claudia Durán, Diana Isabel Pachón-Suárez, Andrés Bustamante-Arias

**Affiliations:** 1 Neuroscience Research Group (NEUROS), Neurovitae Center for Neuroscience, Institute of Translational Medicine (IMT), School of Medicine and Health Sciences, Universidad del Rosario, Carrera 24 # 63C 69, Bogotá, Colombia; 2 Department of Ophthalmology, School of Medicine and Health Sciences, Universidad de Antioquia, Medellín, Colombia; 3 Department of Ophthalmology, School of Medicine and Health Sciences, Universidad Pontificia Javeriana, Cali, Colombia; 4 Department of Ophthalmology, School of Medicine and Health Sciences, FOSCAL, Centro Oftalmológico Virgilio Galvis, Universidad Industrial de Santander, Universidad Autónoma de Bucaramanga, Floridablanca, Santander, Colombia; 5 Ocular Immunology and Uveitis Department, School of Medicine and Health Sciences, Universidad CES, Medellín, Colombia; 6 Clínica de Oftalmología Sandiego, Medellín, Colombia

**Keywords:** Uveitis, Classification, Epidemiology, Colombia, Toxoplasmosis

## Abstract

**Purpose:**

To describe the epidemiology, clinical features, and classification of uveitis in a large cohort of Colombian patients.

**Methods:**

Data were collected from seven ophthalmological referral centers in the four main cities in Colombia. The study included patients with a confirmed diagnosis of uveitis from January 2010 to December 2022. Information on demographics, ophthalmic examination findings, uveitis classification, and etiology was recorded.

**Results:**

The study reviewed 3,404 clinical records of patients with uveitis. The mean age at diagnosis was 41.1 (SD 19.0) years, and 54.2% of the patients were female. Overall, 1,341(39.4%) were infectious, 626 (18.4%) non-infectious, and four masquerade syndromes (0.1%). The most common types of uveitis were unilateral (66.7%), acute (48.3%), and non-granulomatous (83%). Anterior uveitis was the most common anatomical localization (49.5%), followed by posterior uveitis (22.9%), panuveitis (22.3%), and intermediate uveitis (5.2%). A diagnosis was established in 3,252 (95.5%) cases; idiopathic was the most common cause (27.7%), followed by toxoplasmosis (25.3%) and virus-associated uveitis (6.4%). The age group between 30 and 50 exhibited the highest frequency of uveitis.

**Conclusion:**

This multicenter study comprehensively describes uveitis characteristics in Colombian patients, providing valuable insights into its demographic and clinical features. The study findings emphasize the need to continue updating the changing patterns of uveitis to improve diagnosis and treatment strategies for diseases associated with intraocular inflammation.


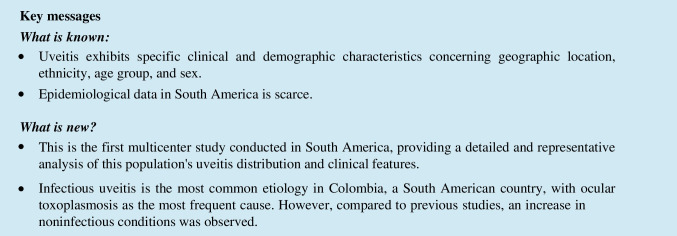


## Introduction

The term “uveitis” encompasses several diseases characterized by intraocular inflammation. It can be infectious or noninfectious, including autoimmune, autoinflammatory, traumatic, post-surgical, drug-induced, and idiopathic. Each type of uveitis has particular demographic and clinical characteristics, but in general, they predominantly affect people of working age, generating a significant economic burden [1–3].

Uveitis prevalence oscillates from 36.2 to 730 per 100,000 inhabitants, and its incidence ranges from 17 to 52.4 per 100,000 inhabitants [4]. These values vary according to the region and the study design that informs them. Similarly, global epidemiological patterns vary due to the influence of several factors, such as environmental, socioeconomic, and epigenetic elements, contributing to different etiologies' prevalence in each region [5–8].

In Colombia, Polania et al. presented the demographic and clinical characteristics of 489 uveitis patients, indicating a notable transition from infectious to immune-mediated etiologies in the last few years as the leading cause of uveitis in the country's capital city [9]. However, data was collected from a single center and may only partially represent the broader reality. Therefore, this multicenter study aims to describe the epidemiology, clinical features, and classification of uveitis in a large cohort of Colombian patients from diverse cities nationwide.

## Methods

### Study design

Multicenter cross-sectional study. It adhered to the Strengthening the Reporting of Observational Studies in Epidemiology (STROBE) guidelines.

### Ethics consideration

The Ethics Committee of Universidad del Rosario approved this study. In addition, this study follows the ethical principles for human research established by the Helsinki Declaration, the Belmont Report, and Colombian Resolution 008430 of 1993.

### Data source and study population

We collected data from seven ophthalmological referral centers across four major cities (Bogotá, Cali, Medellín, and Bucaramanga) and spanning approximately 20 departments within Colombia. The data collection period was extended from January 2010 to December 2022. To ensure the privacy and confidentiality of the patients, all clinical information was procured using a unique identification code, thereby maintaining anonymity. Collected data include demographic details, medical history, specific etiological diagnosis (non-infectious, infectious, undetermined, and idiopathic), visual acuity, uveitis localization, onset, duration, clinical course, and ocular complications (cataract, uveitic glaucoma, macular edema, epiretinal membrane, vitreous hemorrhage, and retinal detachment, among others). Demographic data were primarily acquired during the initial patient visit by administering a questionnaire about uveitis risk factors and systemic symptoms supplemented with a comprehensive physical examination. Ocular findings were collected at the first consultation.

Co-investigators trained in data entry and management were responsible for filling the database to ensure consistency and reliability. Furthermore, two investigators independently verified the accuracy and completeness of the data. Uveitis patients with incomplete or inconsistent data or who were exclusively diagnosed with conditions such as keratitis, optic neuritis, and scleritis were excluded from the dataset (*n* = 92).

### Ophthalmological assessment

Best-corrected visual acuity (BCVA) was measured with Snellen charts, and the values were converted to logarithms of minimal angle of resolution equivalent units (logMAR) for statistical calculations. All patients underwent a comprehensive evaluation by a uveitis specialist, including slit lamp biomicroscopy, intraocular pressure, and dilated fundus examination. Furthermore, clinical data, such as the time of initial uveitis diagnosis, the frequency of uveitis episodes, and laterality, were documented.

### Uveitis definition

Uveitis and anterior chamber grading classification were determined using the Standardization of Uveitis Nomenclature (SUN) criteria [10]. Vitreous haze was graded according to the National Eye Institute system with binocular indirect ophthalmoscopy [11].

Diagnosing systemic disease associated with uveitis was established in a multidisciplinary approach with other medical specialists, including internal medicine, rheumatologists, infectious diseases specialists, and pediatricians. The condition that was the most prominent or more likely related to uveitis was regarded as the primary diagnosis whenever two or more systemic diseases occurred concurrently with uveitis. The diagnosis of *ocular sarcoidosis* was made according to the SUN 2021 revised criteria [12] and the International Workshop on Ocular Sarcoidosis [13]. A chest X-ray was used as the screening tool for chest imaging; computed tomography scanning was used in cases of equivocal chest radiographs or cases with high suspicion of other grounds. Additionally, in instances where sarcoidosis was highly suspected, yet imaging results were inconclusive, it was ruled out using serological tests like Angiotensin-Converting Enzyme levels [14, 15].

Diagnosis of *Blau syndrome* was confirmed by NOD2 mutation [16]. The criteria for a diagnosis of *presumed ocular tuberculosis* included the identification of a tuberculous etiology by Quantiferon Gold TB positivity or Mantoux tuberculin skin test, having or not having abnormalities on chest X-ray, exclusion of other possible causes of uveitis and response to anti-tuberculosis treatment [17]. The diagnosis of the other systemic diseases was determined using the internationally standardized criteria for each disease.

Traumatic iritis was defined as inflammatory cells or flare in the anterior chamber in a patient with recent trauma and in whom infectious and non-infectious uveitis was ruled out. Lens-induced uveitis was defined as an immune reaction to lens material. Moreover, Idiopathic Persistent Iritis after cataract surgery (IPICS) was defined as a transient, non-infectious inflammatory response in the eye that occurs after surgical procedures [18].

Regarding viral uveitis, an initial panel of serology tests was conducted, which included screening for antibodies against Herpes simplex, Herpes zoster, and Cytomegalovirus. When available, patients with atypical presentations underwent aqueous-vitreous humor sampling and a Polymerase Chain Reaction (PCR) [19, 20]. A diagnosis of confirmed virus-associated uveitis was established if a positive result was obtained in PCR. In contrast, in cases where the diagnosis relied only on clinical features and response to antiviral therapy, it was categorized as suspected viral virus-associated uveitis. Furthermore, the diagnosis of ocular toxoplasmosis was made based on clinical criteria, which included positive anti-Toxoplasma IgG and/or IgM test results with an active creamy-white focal retinal lesion with/without hyperpigmented retinochoroidal scars [21].

In cases where an etiology could not be discovered due to a lack of follow-up of the patients, without having ruled out all possible diagnoses, it was considered undetermined. Idiopathic etiology was reserved for cases where the diagnosis could not be determined after ruling out infectious and noninfectious causes of uveitis. It is important to note that while 'Pars planitis' falls under the umbrella of idiopathic conditions, this term was exclusively applied to cases characterized by non-infectious intermediate uveitis accompanied with vitritis and either inferior vitreous inflammatory condensates (“snowballs”) or pars plana “snowbanks”, unassociated with a systemic disease [22].

Uveitis diagnoses were categorized into five groups for analytical clarity and precision. These groups include infectious, non-infectious (encompassing autoimmune, autoinflammatory, and mixed etiologies as delineated by McGonagle and McDermott) [23], masquerade syndromes, idiopathic, and undetermined. Additionally, the category labeled 'others' includes specific etiologies such as traumatic iritis, IPICS, lens-induced uveitis, and drug-induced uveitis.

### Statistical analysis

For the univariate analysis, the continuous variables were reported as mean and standard deviation (SD) or median and interquartile range (IQR) (25th–75th percentile) depending on its distribution, for categorical variables as relative and absolute frequencies and percentages. All the analyses were done using Jamovi (Version 2.3).

## Results

**Fig. 1:**
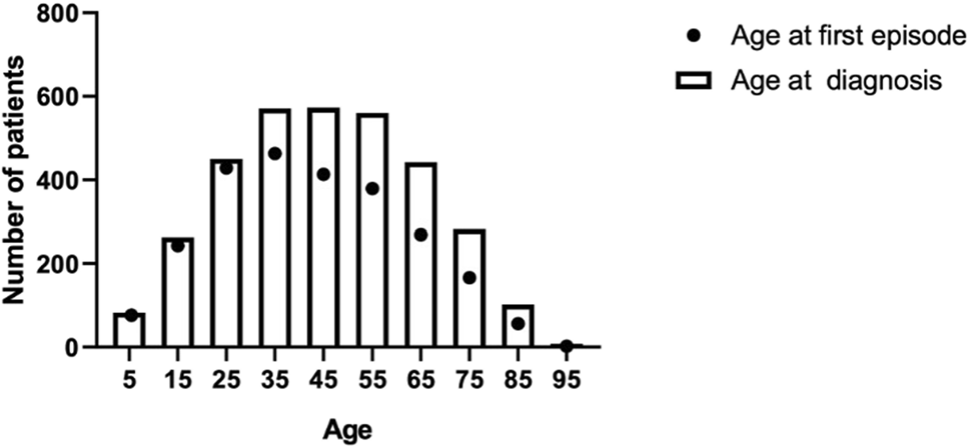
Age distribution of the patients with uveitis in Colombia

**Fig. 2:**
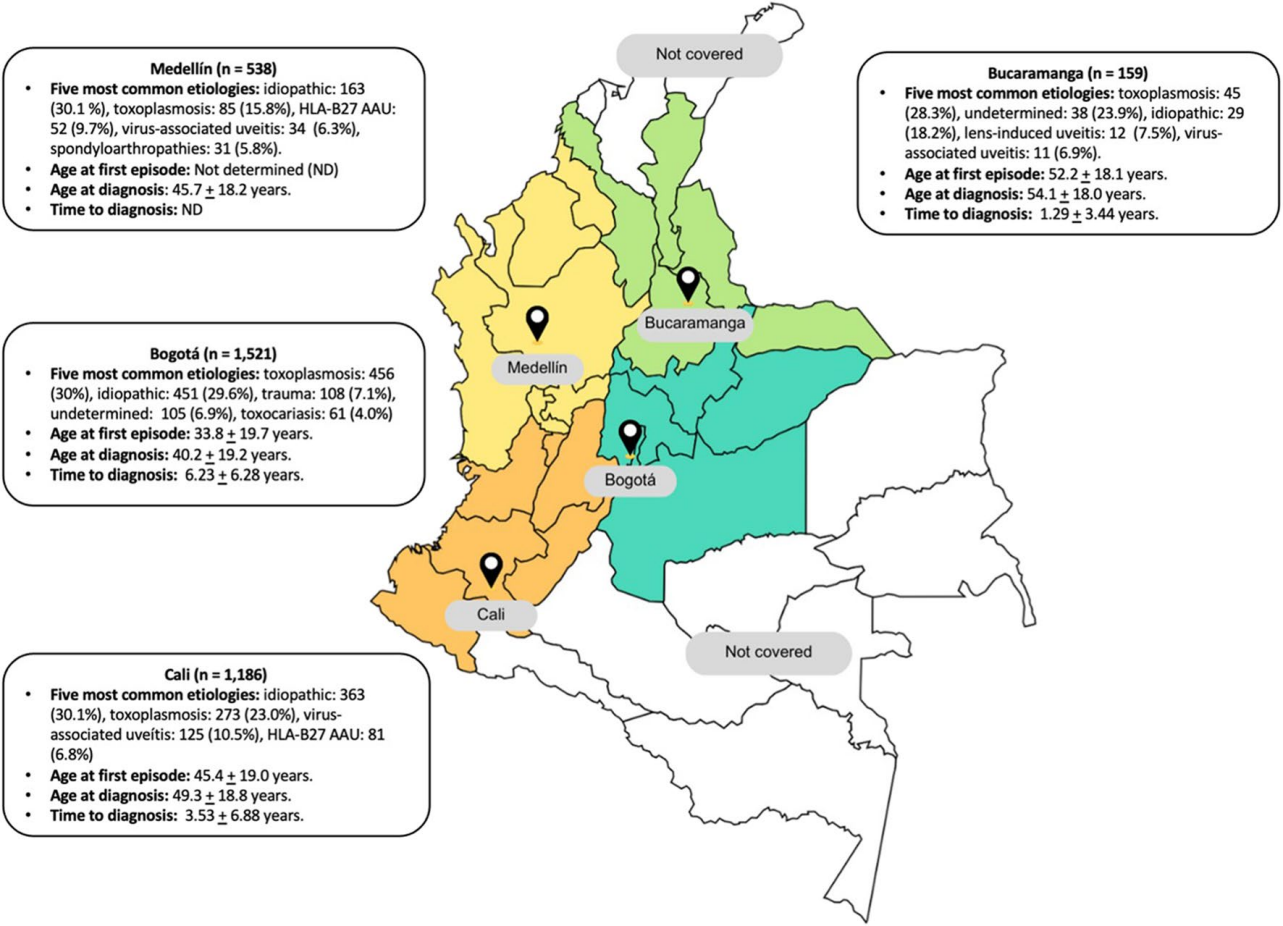
Most common etiologies and ages of onset and diagnosis among the geographical areas covered. Colombian geographical map is divided by departments; yellow represents the areas covered by Medellín centers, orange areas covered by Cali’s centers, green areas covered by Bucaramanga’s centers, and aquamarine areas covered by Bogotá centers. Departments in white are areas where these centers usually do not have coverage. Distribution of main etiologies of uveitis across the different regions

From 3,496 clinical records, we included 3,404 patients diagnosed with uveitis, of which 54.2% (*n* = 1,847) were female. The mean age at onset was 35.7 years, ranging from 1 to 96 years. Overall, 1,341(39.4%) were infectious, 626 (18.4%) non-infectious, and four masquerade syndromes (0.1%). Bilateral involvement was observed in 33% of cases (*n* = 1,124), while 66.7% (*n* = 2,270) presented with unilateral compromise. Table 1, Figs. 1 and 2 show a summary of the demographic information of the included patients.

**Table 1:** Demographic characteristics of patients with uveitis in Colombia

Demographics	Anterior	Intermediate	Posterior	Panuveitis	Total
Age (years) mean ± SD					
At consultation	50.2 ± 17.8	33.5 ± 20.1	38.1 ± 19.5	42.7 ± 18.7	41.1 ± 19.0
At onset	46.2 ± 18.6	29.9 ± 20.8	29.4 ± 20.4	37.4 ± 18.7	35.7 ± 19.6
Gender *n* (%)					
Female	935 (50.6)	98 (5.4)	404 (21.9)	410 (22.2)	1,847 (54.2)
Male	752 (48.6)	78 (5.0)	373 (24.1)	345 (22.3)	1,548 (45.5)
Missing data	0	2 (22.2)	3 (33.3)	4 (44.4)	9 (0.2)
City of Origin *n* (%)					
Bogotá	578 (38.0)	69 (4.5)	452 (29.7)	422 (27.7)	1,521 (44.7)
Bucaramanga	106 (66.7)	0 (0.0)	23 (14.5)	30 (18.9)	159 (4.7)
Cali	697 (58.8)	58 (4.9)	201 (16.9)	230 (19.4)	1,186 (34.8)
Medellín	306 (56.9)	51 (9.5)	104 (19.3)	77 (14.3)	538 (15.8)
Etiology					
Infectious	339 (25.3)	19 (1.4)	634 (47.3)	349 (26.0)	1,341 (39.4)
Non-infectious	411 (65.7)	23 (3.7)	30 (4.8)	162 (25.8)	626 (18.4)
Masquerade	0 (0)	0 (0)	1 (25.0)	3 (75.0)	4 (0.11)
Idiopathic	684 (63.7)	123 (11.5)	77 (7.2)	190 (1.8)	1,074 (31.6)
Undetermined	89 (58.6)	8 (5.3)	18 (11.8)	37 (24.3)	152 (4.5)
Others	152 (80.4)	3 (1.6)	18 (9.5)	16 (8.5)	189 (5.6)
Missing data	12 (66.6)	2 (11.1)	2 (11.1)	2 (11.1)	18 (0.5)
Total *n* (%)	1,687 (49.6)	178 (5.2)	780 (22.9)	759 (22.3)	3,404 (100.0)

In general, anterior uveitis was the most common localization (*n* = 1,687, 49.5%), followed by posterior uveitis (*n* = 780, 22.9%), panuveitis (*n* = 759, 22.3%), and intermediate uveitis (*n* = 178, 5.2%), as evidenced in Table 2. Both anterior and posterior uveitis more frequently presented with an acute course (50.9% and 57.9%, respectively), while intermediate uveitis and panuveitis showed a chronic course in most cases. Of all the anatomical localizations, panuveitis exhibited the highest incidence of complications, including cataracts (*n* = 256, 33.7%), retinal detachment (*n* = 138, 18.2%), and macular edema (*n* = 125, 16.5%). For a more comprehensive overview, please refer to Table 2.

**Table 2:** Characteristics of uveitis in the Colombian population divided by anatomical site of inflammation

Characteristics	Anterior *N* = 1,687	Intermediate *N* = 178	Posterior *N* = 780	Panuveitis *N* = 759	Total *N* = 3,404
Ocular involvement (%)					
Unilateral	1,184 (70.2)	68 (38.2)	569 (72.9)	449 (59.2)	2,270 (66.7)
Bilateral	502 (29.8)	108 (60.7)	208 (26.7)	306 (40.3)	1,124 (33.0)
Missing data	1 (0.1)	2 (1.1)	3 (0.4)	4 (0.5)	10 (0.2)
Course					
Acute	859 (50.9)	55 (30.9)	452 (57.9)	275 (36.2)	1,641 (48.3)
Chronic	421 (25.0)	83 (46.6)	192 (24.6)	329 (43.3)	1,025 (30.1)
Recurrent	400 (23.7)	35 (19.7)	124 (15.9)	146 (19.2)	705 (20.7)
Missing data	7 (0.4)	5 (2.8)	12 (1.5)	9 (1.2)	33 (0.9)
Type of Inflammation					
Non-granulomatous	1,485 (88.0)	150 (84.3)	638 (81.8)	546 (71.9)	2,819 (83.0)
Granulomatous	186 (11.0)	25 (14.0)	130 (16.7)	201 (26.5)	542 (15.9)
Missing data	16 (0.9)	3 (1.7)	12 (1.5)	12 (1.5)	43 (1.2)
Complications at consultation					
Cataract	293 (17.3)	41 (23.0)	77 (9.9)	256 (33.7)	667 (19.7)
Glaucoma	165 (9.8)	14 (7.9)	24 (3.1)	101 (13.3)	304 (9.0)
Macular edema	75 (4.4)	28 (15.7)	35 (4.5)	125 (16.5)	263 (7.8)
Epiretinal membrane	38 (2.3)	17 (9.6)	43 (5.5)	72 (9.5)	170 (5.0)
Vitreous hemorrhage	13 (0.8)	6 (3.4)	21 (2.7)	40 (5.3)	80 (2.4)
Retinal detachment	18 (1.1)	15 (8.4)	64 (8.2)	138 (18.2)	235 (6.9)
Band keratopathy	23 (1.4)	11 (6.2)	4 (0.5)	37 (4.9)	75 (2.2)
Bullous keratopathy	16 (0.9)	3 (1.7)	1 (0.1)	18 (2.4)	38 (1.1)
BCVA (LogMAR)	0.67 ± 0.75	0.76 ± 0.81	1.09 ± 0.93	1.35 ± 1.04	0.96 ± 0.88

*BCVA* Best corrected visual acuity

A specific diagnosis was achieved in 3,252 (95.5%) cases. In 152 (4.5%) of the patients, the cause could not be determined (undetermined uveitis). Overall, idiopathic was the most common cause with 944 patients (27.7%), followed by toxoplasmosis with 858 cases (25.3%) and virus-associated anterior uveitis with 217 cases (6.4%). Regarding gender distribution, females were more commonly affected by idiopathic uveitis than men (60.6% vs 39.4%). On the contrary, men showed a higher prevalence of HLA-B27-associated acute anterior uveitis, accounting for 55.8% compared to 44.2% in women. There was a nearly equal distribution between the sexes for toxoplasmosis, with 433 cases (50.5%) in women and 425 cases (49.5%) in men. (Table 3).

**Table 3:** Laterality and gender distribution of uveitis etiologies in Colombia

Diagnosis	*N* 3404	%	Affected eye		Gender distribution	
			Bilateral *n* (%)	Unilateral *n* (%)	Female *n* (%)	Male *n* (%)
Idiopathic	944	27.73	336 (35.5)	607 (64.3)	572 (60.6)	372 (39.4)
Toxoplasmosis	858	25.34	179 (20.9)	679 (79.1)	433 (50.5)	425 (49.5)
Virus-associated uveitis (confirmed)	217	6.41	18 (8.3)	199 (91.7)	111 (51.2)	106 (48.8)
HLA-B27 Associated Acute Anterior Uveitis	165	4.87	67 (40.6)	98 (59.4)	73 (44.2)	92 (55.8)
Undetermined	152	4.5	48 (31.6)	11 (73.0)	87 (57.2)	72 (47.4)
Pars planitis	130	3.82	80 (61.5)	50 (38.4)	74 (56.9)	56 (43.1)
Traumatic iritis	114	3.37	1 (0.9)	113 (99.1)	21 (18.4)	93 (81.6)
Spondyloarthropathies	83	2.42	39 (47.0)	44 (53.0)	39 (47.0)	44 (53.0)
VKH	73	2.16	0	73 (100.0)	60 (82.2)	13 (17.8)
Toxocariasis	72	2.13	6 (8.3)	66 (91.7)	39 (54.2)	33 (45.8)
Undifferentiated autoinflammatory disease	67	1.98	42 (62.7)	25 (37.3)	42 (62.7)	25 (37.3)
Fuchs uveitis syndrome	52	1.54	14 (26.9)	38 (73.1)	31 (59.6)	21 (40.4)
Rheumatoid arthritis	44	1.30	24 (54.5)	20 (45.5)	37 (84.1)	7 (15.9)
Lens induced uveitis	39	1.15	6 (15.4)	33 (84.6)	19 (48.7)	20 (51.3)
Juvenile Idiopathic Arthritis	37	1.09	29 (78.4)	8 (21.6)	26 (70.3)	11 (29.7)
Syphilis	28	0.83	9 (32.1)	19 (67.9)	15 (53.6)	13 (46.4)
Cytomegalovirus – posterior segment infection	21	0.6	9 (42.9)	12 (57.1)	3 (14.3)	18 (85.7)
Presumed Ocular Tuberculosis	21	0.62	16 (76.2)	5 (23.8)	10 (47.6)	11 (52.4)
Granulomatous polyangiitis	19	0.56	5 (26.3)	14 (73.7)	11 (57.9)	8 (41.0)
SLE	15	0.44	8 (53.3)	7 (46.7)	14 (93.3)	1 (7.7)
Retinitis pigmentosa	15	0.44	13 (86.7)	2 (13.3)	6 (40.0)	9 (60.0)
Sarcoidosis (presumed)	15	0.44	13 (86.7)	2 (13.3)	12 (80.0)	3 (20.0)
Sarcoidosis (definite)	13	0.38	11 (84.6)	2 (15.4)	11 (84.6)	2 (15.4)
Drug-induced uveitis	13	0.38	4 (30.8)	9 (69.2)	13 (100.0)	0
HIV associated	13	0.38	7 (53.8)	6 (46.2)	2 (15.4)	11 (84.6)
Sjögren syndrome	13	0.38	5 (38.5)	8 (61.5)	11 (84.6)	2 (15.4)
Posner-Schlossman Syndrome	12	0.35	3 (25.0)	9 (75.0)	5 (41.7)	7 (58.3)
Behçet's disease	9	0.27	6 (66.7)	3 (33.3)	4 (44.4)	5 (55.6)
Endophthalmitis (acute)	9	0.27	1 (11.1)	8 (88.9)	4 (44.4)	5 (55.6)
Serpiginous Choroidopathy	9	0.24	7 (77.8)	2 (22.2)	4 (44.4)	5 (55.6)
Sympathetic ophthalmia	9	0.27	0	9 (100.0)	5 (55.6)	4 (44.4)
Ulcerative colitis	9	0.27	5 (55.6)	4 (44.4)	6 (66.7)	3 (33.3)
Multiple sclerosis	8	0.24	5 (62.5)	3 (37.5)	6 (75.0)	2 (25.0)
Virus-associated uveitis (suspected)	7	0.21	1 (14.3)	6 (85.7)	3 (42.9)	4 (57.1)
IPICS	6	0.17	1 (16.6%)	5 (83.3)	2 (33.3)	4 (66.6)
Immune recovery uveitis	5	0.15	2 (40.0)	3 (60.0)	2 (40.0)	3 (60.0)
Eales’ disease	4	0.12	2 (50.0)	2 (50.0)	2 (50.0)	2 (50.0)
TINU	4	0.12	3 (75.0)	1 (25.0)	3 (75.0)	1 (25.0)
Birdshot chorioretinopathy	3	0.09	3 (100.0)	0	2 (66.7)	1 (33.3)
Cytomegalovirus – anterior segment infection	3	(0.09)	0	3 (100.0)	1 (33.3)	2 (66.7)
Endophthalmitis (chronic)	3	0.09	1 (33.3)	2 (66.7)	3 (100.0)	0
Histoplasmosis suspected	3	0.09	2 (66.7)	1 (33.3)	1 (33.3)	2 (66.7)
Idiopathic multifocal choroiditis	3	0.09	3 (100.0)	0	2 (66.7)	1 (33.3)
MEWDS	3	0.06	2 (66.7)	1 (33.3)	3 (100.0)	0
Primary intraocular lymphoma	3	0.09	2 (66.7)	1 (33.3)	1 (33.3)	2 (66.7)
Epstein Barr-Virus	2	0.06	1 (50.0)	1 (50.0)	1 (50.0)	1 (50.0)
Multifocal Choroiditis and Panuveitis	2	0.06	2 (100.0)	0	1 (50.0)	1 (50.0)
Psoriasis	2	0.06	1 (50.0)	1 (50.0)	0	2 (100.0)
Relapsing polychondritis	2	0.06	1 (50.0)	1 (50.0)	2 (100.0)	0
UGH syndrome	2	0.06	0	2 (100.0)	0	2 (100.0)
AZOOR	1	0.03	1 (100.0)	0	1 (100.0)	0
Blau syndrome	1	0.03	1 (100.0)	0	0	1 (100.0)
Brucellosis suspected	1	0.03	0	1 (100.0)	0	1 (100.0)
CREST Syndrome	1	0.03	0	1 (100.0)	1 (100.0)	0
Crohn’s disease	1	0.03	1 (100.0)	0	1 (100.0)	0
Cryoglobulinemic vasculitis	1	0.03	0	1 (100.0)	1 (100.0)	0
Cysticercosis	1	0.03	0	1 (100.0)	0	1 (100.0)
IRVAN syndrome	1	0.03	1 (100.0)	0	0	1
Leptospirosis (confirmed)	1	0.03	0	1 (100.0)	0	1 (100.0)
Leptospirosis (suspected)	1	0.03	1 (100.0)	0	0	1 (100.0)
Reactive arthritis	1	0.03	0	1 (100.0)	0	1 (100.0)
Takayasu arteritis	1	0.03	1 (100.0)	0	1 (100.0)	0
Undifferentiated vasculitis	1	0.03	0	1 (100.0)	1 (100.0)	0

*AZOOR* Acute zonal occult outer retinopathy, *IPICS* idiopathic persistent iritis after cataract surgery, *SLE* Systemic lupus erythematosus, *MEWDS* Multiple evanescent white dot syndrome, *TINU syndrome* Tubulointerstitial nephritis and *uveitis syndrome*, *UGH Uveitis-Glaucoma-Hyphema* syndrome, *VKH* Vogt-Koyanagi-Harada disease

In patients diagnosed with anterior uveitis, idiopathic was the most prevalent cause (*n* = 684, 40.8%), followed by virus-associated anterior uveitis (11.9%) (Table 4). On the other hand, in patients with intermediate uveitis, pars planitis remained the leading etiology (*n* = 130, 73%). In posterior uveitis and panuveitis cases, toxoplasmosis was the most prevalent cause (*n* = 548, 70.3%, and *n* = 266, 35%, respectively) (Table 4).

**Table 4:** Causes of uveitis according to the anatomical site of inflammation

Diagnosis	*N* (%)	Anterior *N* = 1, 687 (%)	Intermediate *N* = 178 (%)	Posterior *N* = 780 (%)	Panuveitis *N* = 759 (%)
Idiopathic	944 (27.73)	684 (40.8)	*	77 (9.9)	183 (24.1)
Toxoplasmosis	858 (25.34)	42 (2.5)	3 (1.7)	548 (70.3)	266 (35.0)
Virus-associated uveitis (confirmed)	217 (6.41)	199 (11.9)	3 (1.7)	0	15 (2.0)
HLA-B27 Associated Acute Anterior Uveitis	165 (4.87)	153 (9.1)	7 (3.9)	0	5
Undetermined	152 (4.5)	89 (58.6)	8 (5.3)	18 (11.8)	37 (24.3)
Pars planitis	130 (3.82)	0	130 (73.0)	0	7 (0.9)
Traumatic iritis	114 (3.37)	100 (5.9)	2 (1.1)	4 (0.5)	8 (1.1)
Spondyloarthropathies	83 (2.42)	73 (4.4)	1 (0.6)	0	9 (1.2)
VKH	73 (2.16)	4 (0.2)	1 (0.6)	5 (0.6)	63 (8.3)
Toxocariasis	72 (2.13)	2 (0.1)	3 (1.7)	55 (7.1)	12 (1.6)
Undifferentiated autoinflammatory disease	67 (1.98)	47 (2.8)	2 (1.1)	1 (0.1)	17 (2.2)
Fuchs uveitis syndrome	52 (1.54)	49 (2.9)	2 (1.1)	0	1 (0.1)
Rheumatoid arthritis	44 (1.30)	34 (2.0)	1 (0.6)	0	9 (1.2)
Lens induced uveitis	39 (1.15)	34 (2.0)	0	0	5
Juvenile Idiopathic Arthritis	37 (1.09)	26 (1.6)	2 (1.1)	1 (0.1)	8 (1.0)
Syphilis	28 (0.83)	8 (0.5)	3 (1.7)	5 (0.6)	12 (1.6)
Cytomegalovirus – posterior segment infection	21 (0.6)	0	0	13 (61.9)	8 (38.1)
Presumed Ocular Tuberculosis	21 (0.62)	5 (0.3)	4 (2.2)	2 (0.3)	10 (1.3)
Granulomatous polyangiitis	19 (0.56)	15 (0.9)	0	0	4 (0.5)
SLE	15 (0.44)	11	1 (0.6)	1 (0.1)	2 (0.3)
Retinitis pigmentosa	15 (0.44)	0	0	13 (1.7)	2 (0.3)
Sarcoidosis (presumed)	15 (0.44)	6 (0.4)	3 (1.7)	1 (0.1)	5 (0.7)
Sarcoidosis (definite)	13 (0.38)	1 (0.1)	2 (1.1)	0	10 (1.3)
Drug-induced uveitis	13 (0.38)	10 (0.6)	1 (0.6)	1 (0.1)	1 (0.1)
HIV associated	13 (0.38)	6 (0.4)	0	3 (0.4)	4
Sjögren syndrome	13 (0.38)	10 (0.6)	0	0	3 (0.4)
Posner-Schlossman Syndrome	12 (0.35)	12 (0.7)	0	0	0
Behçet's disease	9 (0.27)	5 (0.3)	0	1 (0.1)	3 (0.4)
Endophthalmitis (acute)	9 (0.27)	4 (0.2)	0	1 (0.1)	4 (0.5)
Serpiginous Choroidopathy	9 (0.24)	0	0	7	2 (0.3)
Sympathetic ophthalmia	9 (0.27)	0	0	2 (0.3)	7 (0.9)
Ulcerative colitis	9 (0.27)	9 (0.5)	0	0	0
Multiple sclerosis	8 (0.24)	2 (0.1)	2 (1.1)	2 (0.3)	2 (0.3)
Virus-associated uveitis (suspected)	7 (0.21)	5 (0.3)	0	0	2 (0.3)
IPICS	6 (0.17)	6 (0.3)	0	0	0
Immune recovery uveitis	5 (0.15)	3 (0.2)	1 (0.6)	0	1 (0.1)
Eales’ disease	4 (0.12)	1 (0.1)	0	3 (0.4)	0
TINU	4 (0.12)	2 (0.1)	0	0	2 (0.3)
Birdshot chorioretinopathy	3 (0.09)	0	0	0	3 (0.4)
Cytomegalovirus – anterior segment infection	3 (0.09)	3 (0.2)	0	0	0
Endophthalmitis (chronic)	3 (0.09)	0	0	1 (0.1)	2 (0.3)
Histoplasmosis suspected	3 (0.09)	1 (0.1)	0	1 (0.1)	1 (0.1)
Idiopathic multifocal choroiditis	3 (0.09)	0	0	0	3 (0.4)
MEWDS	3 (0.06)	0	0	3 (0.4)	0
Primary intraocular lymphoma	3 (0.09)	0	0	1 (0.1)	2 (0.3)
Epstein Barr-Virus	2 (0.06)	0	0	0	2 (0.3)
Multifocal Choroiditis and Panuveitis	2 (0.06)	0	0	0	2 (0.3)
Psoriasis	2 (0.06)	0	0	1 (0.1)	1 (0.1)
Relapsing polychondritis	2 (0.06)	2 (0.1)	0	0	0
UGH syndrome	2 (0.06)	2 (0.1)	0	0	0
AZOOR	1 (0.03)	0	0	1 (0.1)	0
Blau syndrome	1 (0.03)	0	0	0	1 (0.1)
Brucellosis suspected	1 (0.03)	1 (0.1)	0	0	0
CREST Syndrome	1 (0.03)	1 (0.1)	0	0	0
Crohn’s disease	1 (0.03)	1 (0.1)	0	0	0
Cryoglobulinemic vasculitis	1 (0.03)	1 (0.1)	0	0	0
Cysticercosis	1 (0.03)	0	0	1 (0.1)	0
IRVAN syndrome	1 (0.03)	0	0	1 (0.1)	0
Leptospirosis (confirmed)	1 (0.03)	1 (0.1)	0	0	0
Leptospirosis (suspected)	1 (0.03)	0	1 (0.6)	0	0
Reactive arthritis	1 (0.03)	1 (0.1)	0	0	0
Undifferentiated vasculitis	1 (0.03)	1 (0.1)	0	0	0

*AZOOR* Acute zonal occult outer retinopathy, *IPICS* idiopathic persistent iritis after cataract surgery, *SLE* Systemic lupus erythematosus, *MEWDS* Multiple evanescent white dot syndrome, *TINU syndrome* Tubulointerstitial nephritis and *uveitis syndrome*, *UGH syndrome Uveitis-Glaucoma-Hyphema*-syndrome, *VKH* Vogt-Koyanagi-Harada disease, *HIV* Human immunodeficiency virus, *HLA-B27* Human leukocyte antigen B27 *see pars planitis

The age group between 30 and 50 years exhibited the highest prevalence of uveitis, regardless of uveitis localization. In the younger population (< 20 years), posterior uveitis was the most common localization, ranging from 35.5% to 44.6%. Conversely, among individuals over 60 years, anterior uveitis emerged as the most prevalent anatomical localization, ranging from 60.7% to 68.8%. Regarding specific diagnoses, toxoplasmosis was the most frequent in individuals under 40 (26.5% to 44%), while idiopathic cases were more common in those over 40 (29.1% to 49.1%). For detailed data, please refer to Tables 5, 6.

**Table 5:** Uveitis anatomical distribution regarding age groups

Age group	Anterior *N* = 1,687	Intermediate *N* = 178	Posterior *N* = 780	Panuveitis *N* = 759
0–9	12 (14.5)	12 (14.5)	37 (44.6)	22 (26.5)
10.-19	64 (24.2)	51 (19.2)	94 (35.5)	56 (21.1)
20–29	143 (31.8)	22 (4.9)	167 (37.1)	118 (26.2)
30–39	252 (43.9)	23 (4.0)	147 (25.6)	152 (26.5)
40–49	337 (56.4)	24 (4.2)	105 (18.2)	123 (21.3)
50–59	348 (59.8)	17 (3.0)	91 (16.1)	119 (21.1)
60–69	269 (60.7)	19 (4.3)	71 (16.0)	84 (19.0)
70–79	185 (65.6)	6 (2.1)	43 (15.2)	48 (17.0)
> 80	77 (68.8)	1 (0.9)	16 (14.3)	18 (16.1)

*48 patients had not reported their age in the clinical record

**Table 6:** Causes of uveitis by age in Colombia

Diagnosis	0–9	10–19	20–29	30–39	40–49	50–59	60–69	70–79	> 80
	*N* = 83	*N* = 265	*N* = 450	*N* = 574	*N* = 589	*N* = 575	*N* = 443	*N* = 282	*N* = 112
Idiopathic	15 (1.6)	47 (5.0)	99 (10.5)	139 (14.7)	160 (17.0)	160 (17.0)	143 (15.1)	118 (12.5)	54 (5.7)
Toxoplasmosis	22 (26.5)	88 (33.2)	198 (44.0)	197 (34.3)	119 (20.6)	102 (18.1)	74 (16.7)	40 (14.2)	12 (10.7)
Virus-associated uveitis (confirmed)	1 (1.2)	8 (3.0)	20 (4.4)	34 (5.9)	36 (6.2)	39 (6.9)	42 (9.5)	24 (8.5)	11 (9.8)
HLA-B27 Associated Acute Anterior Uveitis	2 (2.4)	4 (1.5)	15 (3.3)	30 (5.2)	42 (7.3)	40 (7.1)	19 (4.3)	9 (3.2)	2 (1.8)
Undetermined	2 (2.4)	7 (2.6)	7 (1.6)	24 (4.2)	25 (4.2)	29 (5.0)	24 (5.4)	22 (7.8)	4 (3.5)
Pars planitis	10 (13.0)	42 (32.3)	19 (14.6)	18 (13.8)	17 (13.1)	8 (6.2)	11 (8.5)	3 (2.3)	1 (0.8)
Traumatic iritis	1 (1.2)	6 (2.3)	19 (4.2)	26 (4.5)	33 (5.6)	14 (2.5)	8 (1.8)	4 (1.4)	3 (2.7)
Spondyloarthropathies	0	0	7 (1.6)	11 (1.9)	26 (4.5)	17 (3.0)	16 (3.6)	6 (2.1)	0
VKH	0	3 (1.1)	10 (2.2)	10 (1.7)	19 (3.3)	19 (3.4)	7 (1.6)	3 (1.1)	0
Toxocariasis	12 (14.5)	26 (9.8)	17 (3.8)	8 (1.4)	5 (0.9)	3 (0.5)	0	1 (0.4)	0
Undifferentiated autoinflammatory disease	1 (1.2)	6 (2.3)	1 (0.2)	13 (2.3)	12 (2.1)	18 (3.2)	13 (2.9)	3 (1.1)	0
Fuchs uveitis syndrome	0	0	1 (0.2)	11	11 (1.9)	16 (2.8)	6 (1.4)	4 (1.4)	3 (2.7)
Rheumatoid arthritis	0	0	1 (0.2)	1 (0.2)	2 (0.3)	3 (0.5)	3 (0.7)	3 (1.1)	2 (1.8)
Lens induced uveitis	0	0	0	3 (0.5)	4 (0.7)	7 (1.2)	9 (2.0)	13 (4.6)	3 (2.7)
Juvenile Idiopathic Arthritis	10 (12.0)	17 (6.4)	7 (1.6)	0	2 (0.3)	1 (0.2)	0	0	0
Syphilis	0	0	3 (0.7)	3 (0.5)	5 (0.9)	8 (1.4)	4 (0.9)	3 (1.1)	2 (1.8)
Cytomegalovirus – posterior segment infection	1 (1.2)	0	1 (0.2)	7 (1.2)	8 (1.4)	4 (0.7)	0	0	0
Presumed Ocular Tuberculosis	2 (2.4)	0	3 (0.7)	2 (0.3)	4 (0.7)	3 (0.5)	6 (1.4)	1 (0.4)	0
Granulomatous polyangiitis	0	0	0	2 (0.3)	3 (0.5)	5 (0.9)	4 (0.9)	2 (0.7)	3 (2.7)
SLE	0	0	0	0	2 (0.3)	2 (0.4)	5 (1.1)	3 (1.1)	0
Retinitis pigmentosa	0	0	0	0	0	1 (0.2)	1 (0.2)	0	0
Sarcoidosis (presumed)	1 (1.2)	2 (0.8)	0	2 (0.3)	1 (0.2)	3 (0.5)	3 (0.7)	0	1 (0.9)
Sarcoidosis (definite)	0	0	0	4 (0.7)	9 (1.6)	12 (2.1)	14 (3.2)	4 (1.4)	1 (0.9)
Drug-induced uveitis	0	0	0	2 (0.3)	0	7 (1.2)	4 (0.9)	0	0
HIV associated	0	0	2 (0.4)	3 (0.5)	2 (0.3)	4 (0.7)	2 (0.5)	0	0
Sjögren syndrome	0	0	1 (0.2)	1 (0.2)	0	3 (0.5)	3 (0.7)	0	1 (0.9)
Posner-Schlossman Syndrome	0	0	0	0	1 (0.2)	0	0	2 (0.7)	1 (0.9)
Behçet's disease	0	0	2 (0.4)	3 (0.5)	0	4 (0.7)	0	0	0
Endophthalmitis (acute)	0	0	1 (0.2)	0	0	2 (0.4)	2 (0.5)	2 (0.7)	2 (1.8)
Serpiginous Choroidopathy	1 (1.2)	3 (1.1)	1 (0.2)	1 (0.2)	3 (0.5)	1 (0.2)	2 (0.5)	2 (0.7)	1 (0.9)
Sympathetic ophthalmia	0	0	0	1 (0.2)	2 (0.3)	4 (0.7)	1 (0.2)	1 (0.4)	0
Ulcerative colitis	0	0	0	2 (0.3)	2 (0.3)	1 (0.2)	2 (0.5)	1 (0.4)	1 (0.9)
Multiple sclerosis	0	0	1 (0.2)	1 (0.2)	0	0	0	0	0
Virus-associated uveitis (suspected)	0	1 (0.4)	1 (0.2)	0	1 (0.2)	2 (0.4)	1 (0.2)	0	0
IPICS	0	2 (0.5)	0	0	0	0	2 (0.5)	0	2 (0.5)
Immune recovery uveitis	0	0	0	1 (0.2)	3 (0.5)	1 (0.2)	0	0	0
Eales’ disease	0	0	1 (0.2)	0	1 (0.2)	1 (0.2)	1 (0.2)	0	0
TINU	1 (1.2)	1 (0.4)	0	0	1 (0.2)	0	1 (0.2)	0	0
Birdshot chorioretinopathy	0	0	0	0	1 (0.2)	0	0	2 (0.7)	0
Cytomegalovirus – anterior segment infection	0	0	0	2 (0.3)	0	0	0	1 (0.4)	0
Endophthalmitis (chronic)	0	0	0	0	0	1 (0.2)	1 (0.2)	1 (0.4)	0
Histoplasmosis suspected	0	0	1 (0.2)	0	1 (0.2)	0	0	0	1 (0.9)
Idiopathic multifocal choroiditis	0	0	0	1 (0.2)	0	2 (0.4)	0	0	0
MEWDS	0	1 (0.4)	0	2 (0.3)	3 (0.5)	5 (0.9)	2 (0.5)	1 (0.4)	1 (0.9)
Primary intraocular lymphoma	0	0	1 (0.2)	1 (0.2)	3 (0.5)	6 (1.1)	1 (0.2)	0	0
Epstein Barr-Virus	0	0	0	1	0	0	1 (0.2)	0	0
Multifocal Choroiditis and Panuveitis	0	0	1 (0.2)	1 (0.2)	0	0	1 (0.2)	0	0
Psoriasis	0	0	0	0	1 (0.2)	1 (0.2)	0	1 (0.4)	0
Relapsing polychondritis	0	0	0	0	1 (0.2)	0	0	0	0
UGH syndrome	0	0	0	0	0	1 (0.2)	0	1 (0.4)	0
AZOOR	0	0	0	0	0	1 (0.2)	0	0	0
Blau syndrome	1 (1.2)	0	0	0	0	0	0	0	0
Brucellosis suspected	0	0	1 (0.2)	0	0	0	0	0	0
CREST Syndrome	0	0	0	0	0	0	1 (0.2)	0	0
Crohn’s disease	0	0	0	0	0	1 (0.2)	0	0	0
Cryoglobulinemic vasculitis	0	0	0	1	0	0	0	0	0
Cysticercosis	0	1 (0.4)	0	0	0	0	0	0	0
IRVAN syndrome	0	0	1 (0.2)	0	0	0	0	0	0
Leptospirosis (confirmed)	0	0	1 (0.2)	0	0	0	0	0	0
Leptospirosis (suspected)	0	1 (0.4)	0	0	0	0	0	0	0
Reactive arthritis	0	0	1 (0.2)	0	0	0	0	1 (0.4)	0
Takayasu arteritis	0	0	0	0	1 (0.2)	0	0	0	0
Undifferentiated vasculitis	0	0	0	1 (0.2)	0	0	0	0	0

*AZOOR* Acute zonal occult outer retinopathy, *IPICS* idiopathic persistent iritis after cataract surgery, *SLE* Systemic lupus erythematosus, *MEWDS* Multiple evanescent white dot syndrome, *TINU syndrome* Tubulointerstitial nephritis and *uveitis syndrome*, *UGH syndrome Uveitis-Glaucoma-Hyphema*-syndrome, *VKH* Vogt-Koyanagi-Harada disease, *HIV* Human immunodeficiency virus, *HLA-B27* Human leukocyte antigen B27

## Discussion

In Colombia, two prior studies have focused on the epidemiology of uveitis. The first was conducted in 2009, encompassing 693 patients from two centers in Bogotá [24]. The second was conducted in 2023 by Polania et al., evaluating 489 patients from a single private center in Bogotá [9]. In the current study, patients from the Polania et al. study were incorporated and combined with six other referral centers from various Colombian cities.

In accordance with prior literature, the demographic group most significantly affected is working-age individuals [25, 26]. The mean age at uveitis diagnosis in our study population was 41.1 years, closely mirroring the findings of a previous single-center report [9]. However, a notable divergence exists in the mean duration between symptom onset and diagnosis, with the current study reporting 5.4 years compared to the previous report's 3.7 years. This discrepancy is concerning, given the substantial burden that delayed treatment initiation can impose in cases of uveitis.

The significant delay between the onset of uveitis symptoms and its diagnosis in Colombia may be attributed to the scarcity of uveitis specialists in the country. This leads to initial referrals of uveitis patients to general ophthalmologists or retina specialists, ultimately resulting in delayed diagnosis and treatment. The frequency of complications related to anatomical localization of uveitis is presented in Table 2.

Regional differences were observed in the time from the initial uveitis presentation to diagnosis. Cali reported an average diagnosis time of 3.53 ± 6.88 years, while Bucaramanga had a lower average of 1.29 ± 3.44 years. Conversely, Bogotá exhibited a considerably a higher diagnosis time of 6.23 ± 6.28 years. This divergence can be attributed to the fact that Bogotá is the most populated city, where the waiting time to be attended could be longer due to high demand and low number of specialists [27].

Although Polania et al. found a higher incidence of uveitis among females [9], the present study shows similar proportions between males and females, similar to what other studies have reported [28]. Including more centers in this study contributes to the heightened representability of uveitis characteristics, which could account for the observed sex differences [29, 30]. Specifically, our study found a higher occurrence of idiopathic uveitis in females (60%). This observation suggests a potential link to the hypothesis that idiopathic uveitis may be influenced by underlying non-infectious conditions and the role of female hormones in stimulating autoimmune responses where inflammation plays a vital role [31]. However, further comprehensive research is essential to fully understand and substantiate these associations.

In the 2009 study, the prevailing characteristics of uveitis included a predominance of unilateral cases (73.4%), an acute clinical course (68.3%), and a non-granulomatous nature (90.6%) [24]. In contrast, Polania et al. in 2023 reported a shift with a higher prevalence of bilateral involvement (52.8%), recurrent presentations (47.6%), and a persistent non-granulomatous pattern (90.8%) [9]. In the current study, the most frequently encountered type of uveitis remains unilateral (66.7%), presenting acutely (48.3%) and maintaining a non-granulomatous nature (83.0%). These consistent features are typically associated with idiopathic uveitis, which emerged as the most prevalent etiology in our study.

Regarding the specific diagnoses, idiopathic uveitis emerged as the most prevalent cause, with toxoplasmosis occupying the second position and virus-associated uveitis ranking third. This pattern of idiopathic uveitis as the predominant etiology is consistent with findings from studies conducted in developed and developing countries [32–38]. This study reinforces the transition in the predominant causes of uveitis in Colombia from infectious to immune-mediated etiologies [9].

Ocular toxoplasmosis remains one of the major causes of uveitis in South America. This is expected due to the higher seroprevalence of *Toxoplasma gondii* in South American countries (45.2%) compared to other regions like Europe (30%) or Western Pacific (11.2%) [39]. Also, clinical presentation tends to be more severe than in other regions [40, 41]. In Colombia, 47.1% of the population have positive IgG titers against *Toxoplasma gondii* and 10.5% of the population have reticochoroidal scars [42]. A nationwide population-based study, found an increasing trend in toxoplasmosis incidence between 2015 and 2019 [43]. This highlights the importance of ongoing patient education about *Toxoplasma gondii* infection. In this context, adhering to practical clinical guidelines is crucial [44].

For anterior uveitis, idiopathic etiology remained the primary cause, followed by virus-related etiologies such as Herpes simplex or zoster and HLA-B27-associated uveitis. Other studies in Colombia similarly report idiopathic uveitis as the leading cause [9]. The higher prevalence of virus-related uveitis in the present study compared to the study conducted by Polania et al. in 2023 could be attributed to the multicenter nature of the current study. This study included several geographic regions, each with differing prevalence of viral diseases, potentially influencing the observed increase [45].

For posterior uveitis and panuveitis, toxoplasmosis remained the primary cause; numerous factors contribute to this, including the Colombian geographical localization in a tropical area with high rainfall (pluviosity) [46] and the presence of certain strains with virulence factors like rhoptry virulent-alleles of proteins (ROP) 16 and ROP 18 [40, 41].

Intermediate uveitis was the least common localization of uveitis, accounting for only 5.2% of the cases, and it was most frequently associated with an idiopathic etiology (pars planitis); this aligns with the typical pattern observed in intermediate uveitis epidemiological data [32, 33, 35–38, 47]. Some studies report diseases like sarcoidosis, multiple sclerosis, and intraocular lymphoma as possible causes of intermediate uveitis [25, 48]. Although these diseases were present in our cohort, they did not represent a significant number of intermediate uveitis [4]. Etiological diagnosis of intermediate uveitis varies between age groups. In children, Pars planitis accounts for most of the cases; this was also observed in this study [49, 50]. Conversely, in older populations (> 70 years old), suspicion of other etiologies like Primary intraocular lymphoma (PIOL) must be considered; [51] the setting of molecular and pathological diagnosis greatly influences the rate of lymphoma detection, in this cohort, PIOL presents as posterior inflammation (1 case) or panuveitis (2 cases). However, we acknowledge the setting of molecular and pathological diagnosis greatly influences the rate of lymphoma detection [52].

Regarding regional variances, anterior uveitis prevailed as the primary localization in Bucaramanga, Cali, and Medellín, in line with global literature [53–55]. However, in Bogotá, posterior uveitis was the most frequently observed, accounting for 29.7% of cases. This divergence can be attributed to the specialization of one of the Bogotá centers in treating toxoplasmosis.

In patients under 16 years, posterior uveitis was the most frequent localization, a pattern attributed to toxoplasmosis being the primary etiology in this age group. As age increased, the frequency of anterior uveitis rose, reaching a peak in the age group between 40 and 60 years, after which it decreased. In the 2009 Colombian study, panuveitis was the most common localization in young and middle-aged adults (16–50 years) [24]. However, the study of Polanía et al. also evidences a change to anterior uveitis [9]. This may be attributed to an increase in idiopathic cases where anterior uveitis was the most common localization and a greater capacity to perform the test for HLA-B27 + identification.

In patients over 50 years old, anterior uveitis remained the predominant localization [9]. This could be associated with increased idiopathic cases and virus-associated uveitis, where anterior uveitis was more commonly observed. Specifically, the incidence of Herpes zoster ophthalmicus in Colombia increased from 0.85 to 1.35 per 100,000 persons between 2015 and 2019, with people over 50 most affected [45].

Compared with other multicenter, population-based studies worldwide, our findings align with the observation that working-age females are predominantly affected, especially for non-infectious conditions. In a study encompassing 3,000 patients in the UK, Jones identified a clear preference for the female gender in cases of Juvenile idiopathic arthritis-associated uveitis (78%), Punctate Inner Choroidopathy (76%), and Chronic Anterior Uveitis (62%) [56]. In our analysis, females significantly outnumbered males in conditions such as Multiple evanescent white dot syndrome (100%), Vogt-Koyanagi-Harada syndrome (82.2%), Multiple Sclerosis (75%), and idiopathic etiology (60%). Conversely, males showed a higher prevalence of trauma iritis (81.6%), IPICS (66%), and infectious causes like Human Immunodeficiency Virus (89.6%) and Acute Retinal Necrosis (78.8%). Different factors could contribute to these differences, including genetics, sex hormones, and social factors [57–59].

Anterior uveitis is the more prevalent localization in both the Western [53, 56] and Eastern world, [54, 55], with idiopathic etiology leading the list, trailed by infectious and non-infectious [54]. However, distinct patterns emerge in certain developing countries. For instance, Brazil reported a deviation from this trend, with posterior uveitis accounting for most cases (43.1%); [26], although this was observed in 1,053 patients, they were all from a single center. Similarly, in Colombia, posterior uveitis was the most common localization decades ago [24]. Nevertheless, this study noted a recent increase in cases of anterior uveitis. This shift is explained by the increase in idiopathic cases, which were commonly anterior.

To rule out an infectious etiology is a pivotal step of uveitis diagnosis and treatment, then it is essential to know the main etiologies according to the geographical prevalence. In South America, toxoplasmosis remains the predominant infectious cause, with viral anterior uveitis following closely. This aligns with other South American countries where toxoplasmosis accounts for 24.03% of all uveitis cases [26]. However, it differs from Asian countries where ocular tuberculosis is more prevalent compared to our population (19.6% vs. 0.3%) [54]. Other infections, such as syphilis, although not the most common, must always be ruled out [55].

The main strength of this study is the multicenter methodology across different cities in Colombia, providing a comprehensive description of uveitis. Prior research has often relied on small samples from tertiary centers or focused solely on specific causes, limiting their generalizability. Our study includes seven referral centers from four main cities in Colombia, providing a comprehensive representation of patients from the central regions of the country (where most of the population is located) (Fig. 2). Uveitis is generally an alarming entity that forces patients to consult and in most cases general ophthalmologists refer patients to the uveitis specialist, so we estimate that many of the patients who live in cities other than the evaluated surely consulted in one of these centers, which reduces the potential loss of cases.

Our study has some limitations. Due to economic and accessibility disparities among the country's regions, the potential for selection bias cannot be entirely ruled out. Nonetheless, by encompassing private and public centers in our study, we aimed to reduce this risk, considering the extensive coverage reported by the Colombian health system (97.78%). Furthermore, as our data was gathered from tertiary referral centers, there is a potential for overestimating rarer etiologies and underestimating cases of uveitis that are easily treatable. Moreover, previous Colombian studies reported the prevalence of an undetermined etiology at around 16.5% and 21.6%, but in our study, it was only 4.5% [9, 24]. Therefore, we acknowledge the possibility that some undetermined cases could have been mistakenly categorized as idiopathic. However, given that experienced uveitis specialists conducted most diagnoses, and the data was meticulously recorded by knowledgeable personnel following the SUN criteria, the likelihood of this risk is minimized. Moreover, we report diagnoses like rheumatoid arthritis, systemic lupus erythematosus, and antiphospholipid syndrome. However, these diseases should be understood as possible systemic associated conditions to uveitis and not as a direct cause [9, 60–63].

In conclusion, this is the most extensive multicenter study conducted in South America, focusing on the epidemiology of uveitis. Infectious uveitis remains the most common etiology, with ocular toxoplasmosis as the most frequent cause. However, compared to previous studies, an increase in non-infectious conditions was also observed. This study emphasizes the importance of conducting multicenter research to identify changes in the patterns of uveitis and keep updated the epidemiological knowledge of these group of diseases.

## Data Availability

The datasets used and analyzed during the current study are available from the corresponding author upon reasonable request.

## Abbreviations

*JIA*: Juvenile idiopathic arthritis
*AZOOR*: Acute zonal occult outer retinopathy
*BCVA*: Best-corrected visual acuity
*HIV*: Human Immunodeficiency Virus
*HLA-B27*: Human leukocyte antigen B27
*IOL*: Intra-ocular lens
*IPICS*: Idiopathic persistent iritis after cataract surgery
*MEWDS*: Multiple evanescent white dot syndrome
*PIOL*: Primary intraocular lymphoma
*SUN*: Standardization of uveitis nomenclature
*STROBE*: Strengthening the reporting of observational studies in epidemiology
*SLE*: Systemic lupus erythematosus
*TINU syndrome*: Tubulointerstitial nephritis and uveitis syndrome
*UGH*: Uveitis-Glaucoma-Hyphema syndrome
*VKH*: Vogt-Koyanagi-Harada disease
